# Non-enzymatic posttranslational protein modifications in protein aggregation and neurodegenerative diseases

**DOI:** 10.1039/d4cb00221k

**Published:** 2024-12-19

**Authors:** Tim Baldensperger, Miriam Preissler, Christian F. W. Becker

**Affiliations:** a University of Vienna, Faculty of Chemistry, Institute of Biological Chemistry Währinger Str. 38 1090 Vienna Austria tim.baldensperger@univie.ac.at miriam.preissler@univie.ac.at christian.becker@univie.ac.at; b University of Vienna, Vienna Doctoral School in Chemistry (DoSChem) Währinger Str. 42 1090 Vienna Austria

## Abstract

Highly reactive metabolic intermediates and other small molecules frequently react with amino acid side chains, leading to non-enzymatic posttranslational modifications (nPTMs) of proteins. The abundance of these modifications increases under high metabolic activity or stress conditions and can dramatically impact protein structure and function. Although protein quality control mechanisms typically mitigate the effects of these impaired proteins, in long-lived and degradation-resistant proteins, nPTMs accumulate. In some cases, such as cataract development and diabetes, clear links between nPTMs, aging, and disease progression have been established. In neurodegenerative diseases such as Alzheimer's and Parkinson's disease, a key question is whether accumulation of nPTMs is a cause or consequence of protein aggregation. This review focuses on major nPTMs found on proteins with central roles in neurodegenerative diseases such as α-synuclein, β-amyloid, and tau. We summarize current knowledge on the formation of these modifications and discuss their potential impact on disease onset and progression. Additionally, we examine what is known to date about how nPTMs impair cellular detoxification, repair, and degradation systems. Finally, we critically discuss the available methodologies to systematically investigate nPTMs at the molecular level and outline suitable approaches to study their effects on protein aggregation. We aim to foster more research into the role of nPTMs in neurodegeneration by adapting methodologies that have proven successful in studying enzymatic posttranslational modifications. Specifically, we advocate for site-specific incorporation of these modifications into target proteins using advanced chemical and molecular biology techniques.

## Abstract 2

In this review we summarize the known non-enzymatic posttranslational modifications (nPTMs) of proteins involved in neurodegeneration and discuss illustrative examples of such nPTMs studied using sophisticated protein engineering approaches.

## Graphical abstract

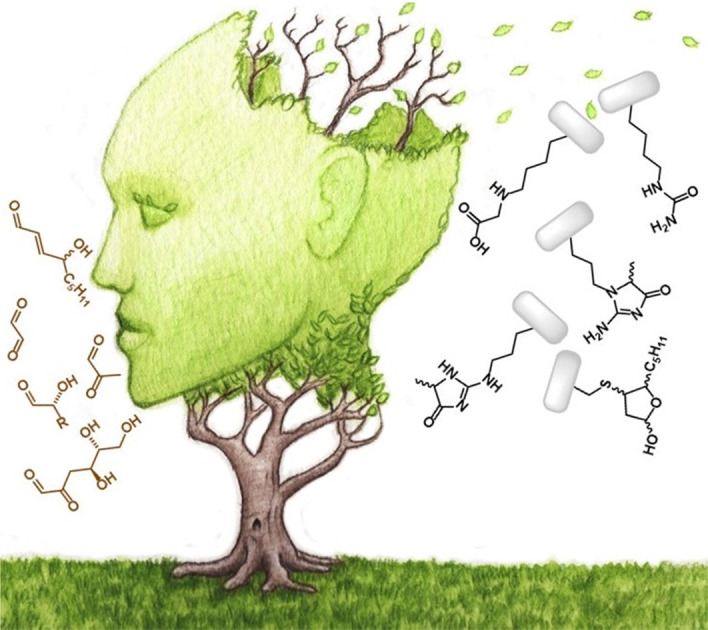

## Introduction

According to the World Health Organization, dementia affects over 55 million people globally, with this number expected to rise to approximately 140 million by 2050. Dementia is the seventh leading cause of death and resulting annual healthcare costs were estimated to be US$ 1.3 trillion in 2019.^1^ The majority of dementia cases are attributed to neurodegenerative diseases such as Alzheimer's disease (AD) and Parkinson's disease (PD).^2^ An unifying hallmark of these pathologies is the accumulation of aggregated proteins.^3^ For the past decades, researchers have intensely debated why functional proteins such as α-synuclein (αSyn), β-amyloid (Aβ), and tau start to aggregate and accumulate during aging and to what extent these aggregates contribute to neurodegeneration.^4–7^

One hypothesis involves the impact of posttranslational modifications (PTMs), which are chemical changes to proteins formed either strictly regulated by enzymes or non-enzymatically due to reactions with metabolites.^8^ The effects of enzymatic PTMs such as phosphorylation, acetylation, and ubiquitylation on protein aggregation, degradation, and resulting cytotoxicity have been extensively studied and reviewed.^9–13^ In stark contrast, the role of non-enzymatic PTMs (nPTMs) is much less understood. This is particularly concerning since nPTMs accumulate with age, are prevalent in diseases associated with increased risk of neurodegeneration, and are highly abundant in protein aggregates of neurodegenerative diseases.^14–19^

Two key processes leading to nPTMs are oxidative and carbonyl stress.^20,21^ Formation of reactive oxygen species (ROS) is an inevitable part of cellular metabolism. For instance, oxygen consuming processes such as electron transport chains, NADPH oxidases, and cytochrome P450 systems generate superoxide anion radicals. The formed superoxide is detoxified to hydrogen peroxide, leading to formation of hydroxyl radicals *via* the transition metal catalyzed Fenton reaction.^22^ Hydroxyl radicals are the most reactive ROS and directly modify proteins as well as other macromolecules leading to secondary ROS formation, *e.g.*, alkoxyl and peroxyl radicals in the course of lipid peroxidation.^21^ Furthermore, superoxide readily reacts with the cellular messenger molecule nitric oxide and produces reactive nitrogen species (RNS) like peroxynitrite, which spontaneously decomposes into nitrogen dioxide and hydroxyl radicals.^23^ When cellular antioxidative and repair systems are overwhelmed by these processes, the resulting oxidative stress leads to accumulation of oxidative nPTMs.^20^ Similarly, the excessive generation of reactive carbonyl species (RCS) is termed carbonyl stress.^24^ Carbonyl stress has a strong overlap with oxidative stress, *e.g.*, RCS such as glyoxal (GO), malondialdehyde, and 4-hydroxy-2-nonenal (4-HNE) are generated by oxidative degradation of lipids.^21^ Energy metabolism is another important source of RCS, for example *via* triosephosphate degradation as the main source of methylglyoxal (MGO)^25^ or formation of reactive acyl-CoA species (RACS) in the citric acid cycle.^26^ Moreover, RCS like deoxyglucosones and their cleavage products are formed from carbohydrates in the Maillard reaction.^27^ These RCS eventually lead to protein modifications known as advanced lipoxidation endproducts (ALEs) and advanced glycation endproducts (AGEs), depending on their metabolic origin.^21^ Interestingly, carbonyl stress is not merely a consequence of oxidative stress, but *vice versa* carbonyl stress is an initiator of oxidative stress by damaging mitochondria^28^ and inducing inflammation.^29^

**Fig. 1:**
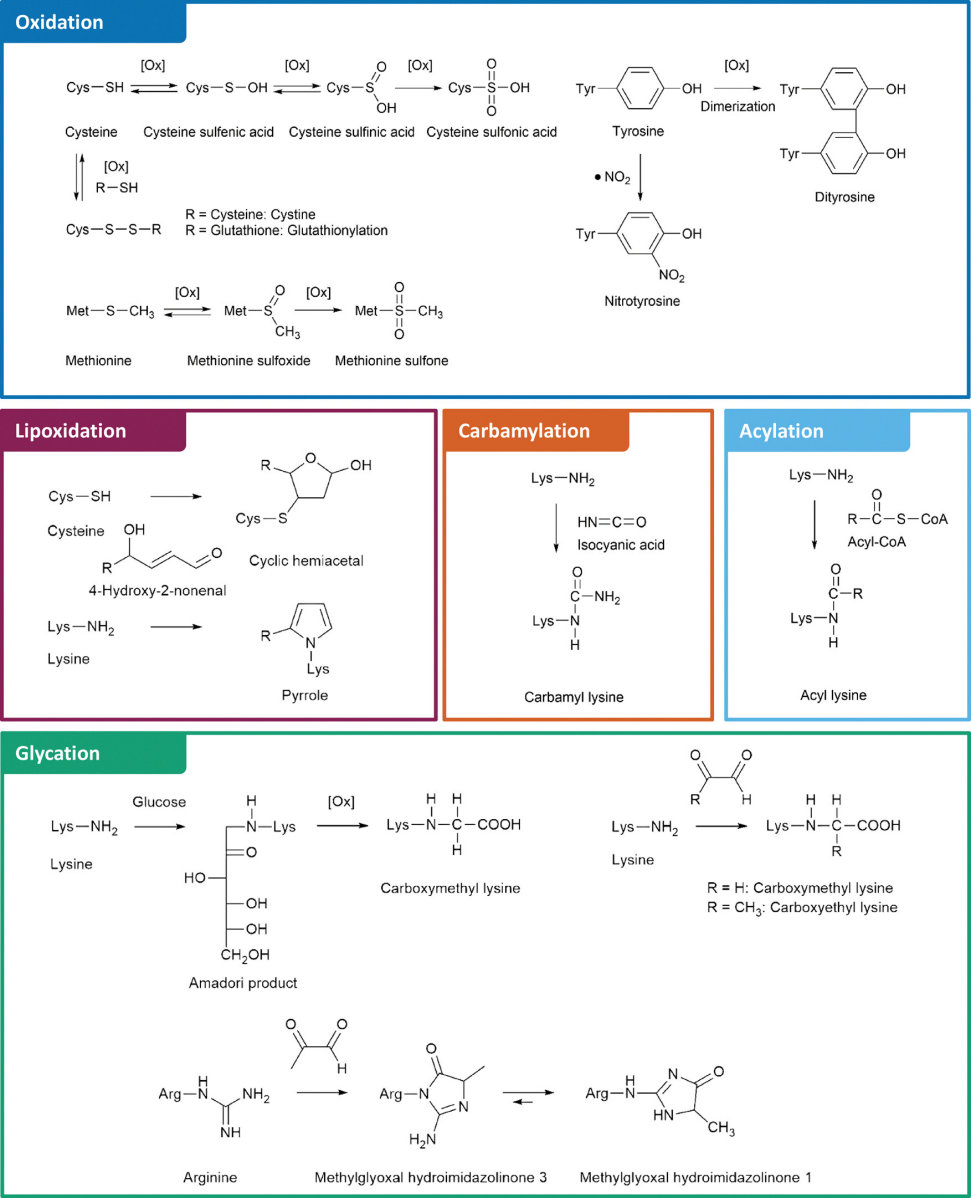
Non-enzymatic posttranslational protein modifications (nPTMs) of protein aggregates.

The structures and formation mechanisms of the most relevant nPTMs in the context of protein aggregation and neurodegeneration are summarized in Fig. 1. Major targets of protein oxidation include cysteine, methionine, and tyrosine residues. Cysteine is readily oxidized by ROS to cysteine sulfenic and sulfinic acid, which is a reversible process by the enzyme glutaredoxin.^30^ Further oxidation to cysteine sulfonic acid is considered as irreversible “overoxidation” and typically prevented by protective glutathionylation.^31^ Alternatively, cysteine is able to form disulfide bonds with a second cysteine moiety under oxidative conditions, which is a fundamentally important mechanism in protein folding and reversible by several enzymes.^32^ Methionine, the second sulfur containing proteinogenic amino acid, is oxidized to methionine sulfoxide. This process is reversible *via* methionine sulfoxide reductases.^33^ In contrast, further oxidation to methionine sulfone is an irreversible step.^34^ Tyrosine oxidation is a potential source of protein crosslinking through dityrosine formation.^35^ Moreover, tyrosine is commonly nitrated by RNS leading to 3-nitrotyrosine.^36^

Beyond direct oxidation of amino acids, several proteins are modified by reactive species originating from oxidative processes. For instance, lipoxidation intermediates such as 4-HNE form various adducts with lysine, cysteine, and histidine residues.^21^ The myeloperoxidase catalyzed oxidation of thiocyanate or spontaneous urea decomposition results in formation of isocyanic acid,^37^ which is the precursor of non-enzymatic lysine carbamylation.^38^ An alternative lysine modification is acylation by RACS.^39^ Several of these reactions are catalyzed by lysine acyl transferases.^40^ However, some RACS such as succinyl-CoA form highly reactive intramolecular anhydride structures leading to efficient formation of nPTMs.^41^

Finally, glycation by the Maillard reaction represents another major pathway of nPTMs, in which a nucleophile, *e.g.*, the amino group of lysine or the guanidino group of arginine reacts with RCS such as deoxyglucosones or MGO. These RCS are central intermediates in glycation cascades and lead to formation of important AGEs, including *N*^6^-carboxymethyl lysine (CML), *N*^6^-carboxyethyl lysine (CEL), and arginine hydroimidazolinones.^42,43^

In this review, we aim to comprehensively summarize the complex network of nPTMs reported so far for protein aggregates associated with neurodegenerative diseases. We place particular emphasis on understanding how these nPTMs contribute to the neurotoxicity of protein aggregates, as well as how nPTMs impair cellular defense mechanisms. Last but not least, we critically evaluate the existing literature, describe approaches to generate selectively nPTM-carrying proteins, identify research gaps and propose future directions to enhance both the research quality and to inspire further investigations in this important field.

### α-Synuclein

**Fig. 2:**
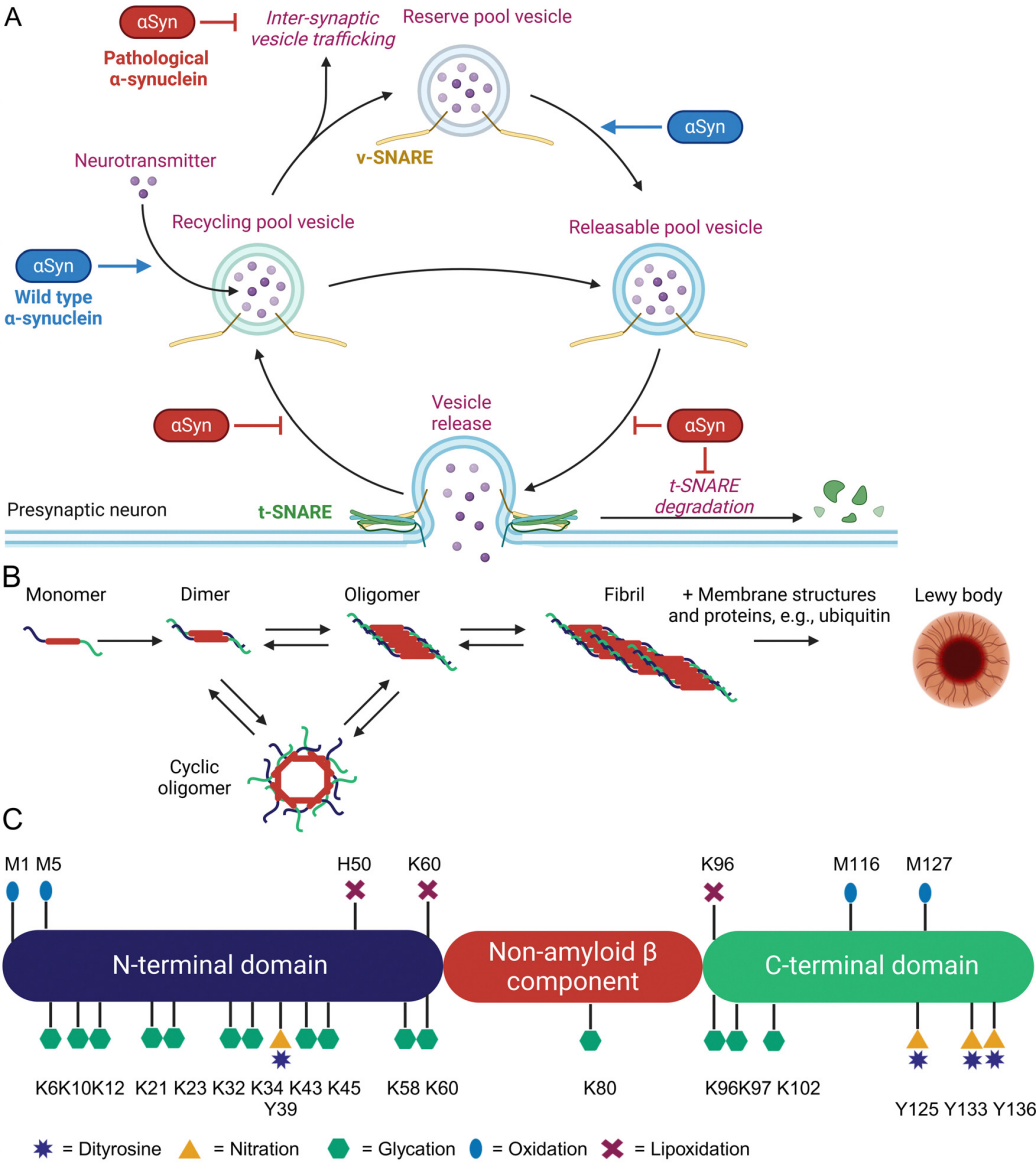
Overview of function and aggregation of α-synuclein, including known non-enzymatic posttranslational modifications. Cellular function and pathogenic effects of α-synuclein (A). Oligomerization, fibrillization, and aggregation of α-synuclein (B). Non-enzymatic posttranslational modifications of α-synuclein (C).

α-Synuclein (αSyn) is highly abundant in the central nervous system, making up approximately 0.5 to 1.0% of cytosolic brain proteins.^44^ It is predominantly located at the presynaptic termini of neurons and colocalized with several proteins responsible for neurotransmitter release and re-uptake of synaptic vesicles.^45^ Despite extensive research, the exact role of αSyn in neurotransmitter shuttling remains controversial.^46^ Knock-out and overexpression experiments suggest a regulatory function, as absence of αSyn caused a lack of synaptic vesicles, while overexpression enhanced the number of available vesicles (Fig. 2(A)).^47^

The 14.4 kDa αSyn protein comprises 140 amino acids and lacks cysteine, arginine, as well as tryptophan residues.^48^ Structurally, αSyn consists of three distinct domains: an amphipathic and lysine-rich amino terminus (residues 1–60), the hydrophobic non-amyloid-β component region (residues 61–95), and a disordered, acidic carboxy-terminal tail (residues 96–140).^49^ These domains form an intrinsically disordered protein,^50^ but upon binding to lipid membranes the N-terminus rearranges into an α-helix of 95 amino acids.^51^ This binding is mediated by repetitive KTKEGV motifs mainly located in the N-terminal region.^52^ The non-amyloid-β component domain is mandatory for aggregation of αSyn through β-sheet formation.^53^ Last but not least, the negatively charged C-terminus has been shown to bind Ca^2+^ and can exhibit chaperone-like activity.^54^ Initially, αSyn was identified as the non-amyloid-β component in amyloid plaques found in the brains of Alzheimer's disease patients.^44^ It is also a major component of protein aggregates known as Lewy bodies and Lewy neurites, which are hallmarks of neurodegenerative diseases termed as synucleinopathies, *e.g.*, Parkinson's disease (PD), dementia with Lewy bodies, multiple systems atrophy, and pure autonomic failure.^47^ Aggregation of αSyn involves several intermediates such as dimers, oligomers, fibrils, and finally Lewy body formation, which additionally contain various proteins (ubiquitin, p62) and lipids (membrane structures) as shown in Fig. 2(B).^55^ Recent research indicates that oligomeric forms of αSyn, rather than its fibrils or aggregates, are the toxic species contributing to cellular toxicity, similar to other pathologic protein aggregation events.^56–58^ These oligomers exert their detrimental effects through several mechanisms including increased Ca^2+^ influx by altered membrane integrity,^59^ mitochondrial damage,^60^ lysosomal leakage,^61^ and disruption of microtubules.^62^ A critical and still unanswered question is, why a functional important protein such as αSyn starts to form toxic protein aggregates. Approximately 5 – 10% of PD patients have a genetic predisposition due to specific mutations in the SNCA gene, which leads to the expression of harmful αSyn variants.^57^ For the remaining PD patients, the onset appears to occur spontaneously. Parkinson's disease is predominantly an age-related condition, rarely diagnosed in individuals under 50 years of age, but affecting approximately 1% of people over 60 years old.^63^ Additional risk factors include exposure to environmental toxins, pesticides, and heavy metals, as well as comorbidities such as head injuries or viral infections. Conversely, lifestyle interventions such as regular physical activity and coffee consumption have been shown to have protective effects.^64^ Type 2 diabetes has also been identified as a risk factor for PD. An English retrospective study involving 8 190 323 individuals demonstrated a 1.3 to 1.5-fold increased risk of PD among diabetics.^65^ Similarly, a Korean follow-up study with 2 362 072 participants found that the hazard rate of developing PD increased between 1.1 and 2.8 times, depending on the severity of diabetes mellitus.^66^

Based on these studies, a common feature of all risk factors in PD is the generation of inflammation, oxidative and carbonyl stress that also leads to enhanced non-enzymatic protein modifications (Fig. 2(C)) at various αSyn sites.^15,20^

One of the most abundant nPTMs in αSyn is oxidation of methionine to methionine sulfoxide.^33,67^ Residue 5 is the most susceptible residue for methionine sulfoxide formation and was already formed by treatment of recombinantly expressed αSyn with UV light. Oxidation of M5 produced fibrilization-incompetent αSyn and favored the formation of neurotoxic oligomers.^68^ Oxidation of M1 and M5 by hydrogen peroxide drastically inhibited αSyn degradation by the 20S proteasome and this effect was nullified in a M1,5A mutant.^69^ Dopamine was able to oxidize M116 and M127 leading to soluble and cytotoxic oligomers, which were rendered harmless by M127A mutagenesis in a cellular test system.^67^ Oxidation of M127 reduces phosphorylation of nearby Y125 and possibly S129, which are important modulators of αSyn aggregation and toxicity.^70^ Oxidation of all 4 methionine residues in αSyn to the sulfoxides dramatically inhibited fibrillization,^71^ an effect which was proportional to the amount of oxidized methionine residues,^72^ and decreased the binding capacity in a model of synaptic vesicles.^73^ Interestingly, methionine oxidation at position M1 and M5 is reversible by methionine sulfoxide reductases, while positions M116 and M127 are not targeted by this repair system.^70^ Reversible oxidation of M1 and M5 is considered as a possible ROS scavenger and protects αSyn from oxidation at more harmful positions. Consequently, over-expression of methionine sulfoxide reductase A prevented development of PD-like symptoms in *Drosophila melanogaster* after ectopic expression of αSyn.^74^ Tyrosine is another common target of protein oxidation in αSyn, which can form intra- and intermolecular dityrosine crosslinks.^75,76^ Intramolecular crosslinking of tyrosine residue 39 with 125, 133, or 136 was reported to prevent αSyn fibrilization and aggregation by stabilizing monomers.^77^ In contrast, intermolecular tyrosine dimerization is a critical and rate limiting step in αSyn aggregation leading to PD.^78^ Tyrosine is also modified by nitroxidative stress. The group of Lashuel utilized native chemical ligation to generate aSyn variants with site-selective nitrotyrosine residues at positions 39 and 125. These variants formed big amorphous aggregates in contrast to long fibrils formed by wild-type αSyn and the nitrated αSyn had a reduced binding affinity for membrane vesicles.^79^ The reduced vesicle binding was mainly caused by Y39 nitration and nitrated αSyn was more resistant towards degradation by the 20S proteasome and calpain.^80^ Despite close proximity, nitration of Y125 had no effect on S129 phosphorylation.^79^ Lipoxidation byproducts, such as 4-HNE, form adducts with histidine H50, lysine residues K60 and K96, which enhanced formation of αSyn oligomers and toxicity in cultured neurons.^81–83^ A distinctive characteristic of αSyn is its modification by dopamine and its oxidized metabolites, such as dopamine quinone and 3,4-dihydroxyphenylacetaldehyde.^84^ Dopaminergic neurons are the primary cell type affected in PD and interactions between αSyn and dopamine have been shown to exacerbate neurotoxicity.^85^

Deposits of αSyn in both mice and humans accumulate AGEs.^86–88^ However, whether the accumulation of AGEs is the cause or merely a consequence of protein aggregation remains an unresolved question. Glycation of recombinant αSyn by GO and MGO induced oligomerization, inhibited fibrilization and resulted in small spherical aggregates.^89^ Glycation by MGO furthermore resulted in loss of binding to anionic lipid membranes.^90^ Extensive modification of all 14 lysine residues to form CEL led to oligomer formation and prevented fibrillization^91^ as well as binding to small vesicles.^92^ Increase of MGO levels by knock-down of glyoxalase 1 or triosephosphate isomerase increased αSyn aggregates and toxicity in yeast, Lund human mesencephalic cells, and *Drosophila melanogaster*.^88^ Intracerebroventricular MGO injection in mice exacerbated PD-like symptoms.^93^ So far, only the group of Fleming-Outeiro has produced selectively CML/CEL-modified αSyn fragments and a full-length version of the PD-associated αSyn variant E46K, with a single CEL modification at position 46. However, the biological evaluation of these modified species remains to be conducted.^94^

### β-Amyloid

**Fig. 3:**
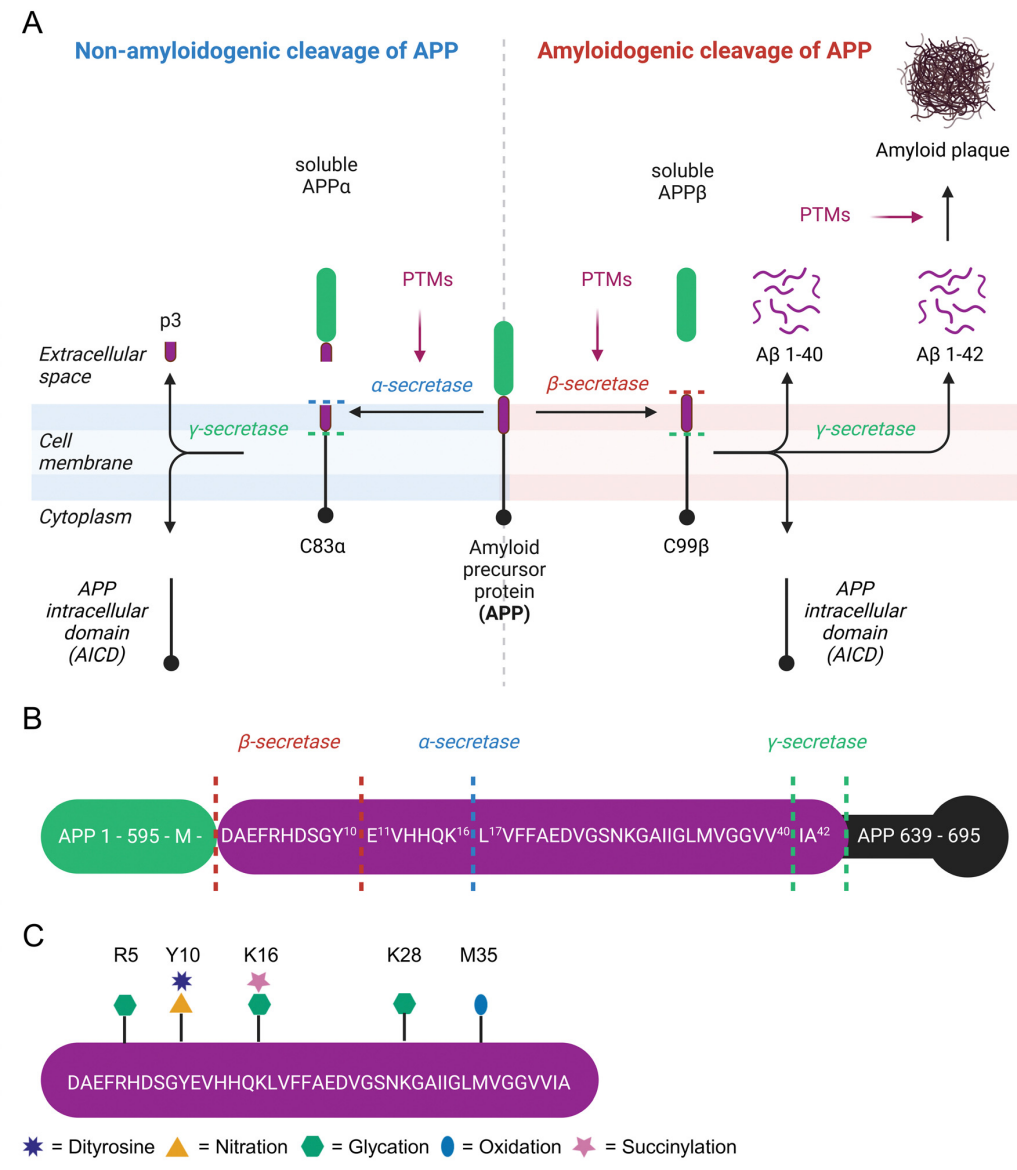
Overview of amyloid-beta, formation of β-amyloid aggregates, and known non-enzymatic posttranslational modification sites. Non-amyloidogenic and amyloidogenic processing of amyloid precursor protein (A). Possible cleavage sites of secretases in the amyloid precursor protein sequence (B). Non-enzymatic posttranslational modifications of β-amyloid 1–42 (C).

β-Amyloid (Aβ) is a 4 kDa fragment originating from proteolytic cleavage of the amyloid precursor protein (APP). This precursor is expressed by brain neurons, astrocytes, vascular and blood cells.^95^ APP has three isoforms APP695, APP751, and APP770 arising from alternative splicing. APP695 is the predominant isoform in the brain and lacks a 56 amino acid Kunitz Protease Inhibitor domain found in APP751 and both the Kunitz Protease Inhibitor domain and a 19 amino acid OX-2 domain present in APP770.^96^ APP is a transmembrane protein that spans the extracellular space from the N-terminus to the intracellular lumen at the C-terminus. It is processed by secretases either in a non-amyloidogenic or an amyloidogenic pathway.^97^ In the non-amyloidogenic pathway, cleavage by α-secretase generates soluble APPα (sAPPα) and the C83α subunit.^98^ C83α is further processed by γ-secretase to form the APP intracellular domain (AICD) and the p3 fragment.^99^ Extracellular sAPPα is a neuroprotective protein and vitally important for cognitive function.^100^ The amyloidogenic pathway involves APP cleavage by β-secretase into soluble APPβ (sAPPβ) and the C99β subunit.^101^ The latter is further processed by γ-secretase to yield AICD and Aβ peptides, which consist of 37 to 43 amino acids.^102^ The major product Aβ 1–40 is about 10 times more abundant in cerebrospinal fluid than Aβ 1–42.^102^ Aβ 1–42 forms intracellular oligomers and is the main component of extracellular amyloid plaques,^103^ which are a hallmark of Alzheimer's disease (AD).^104^ Alternatively, β-secretase has a second APP cleavage site leading to a C89β fragment, which is further processed to Aβ 11–42 and lacks amyloidogenic properties (Fig. 3(A)).^105^

A small portion (<5%) of AD cases are early-onset variants caused by mutations in the APP, PSEN1, or PSEN2 genes.^106^ However, the majority of AD patients is affected by late-onset forms with the disease affecting 1 in 9 individuals over the age of 65 and 1 in 3 individuals over the age of 85, making age the leading risk factor of AD.^107^ The most significant genetic risk factor for late-onset AD is the polymorphism in the apolipoprotein E (ApoE) gene. The ApoE ε4 allele is present in 60% of Caucasian AD patients. The risk of developing AD is 2–3 times higher with one ε4 allele and about 12 times higher in those with two ε4 alleles compared to ApoE ε3.^108^ People with ApoE ε4 polymorphism suffer from impaired glucose metabolism, increased oxidative stress, and enhanced neuroinflammation as potential molecular drivers of AD as reviewed previously.^109^ In contrast, ApoE ε2 is protective against development of AD.^110^ Beside genetic predisposition, various comorbidities contribute to AD development. For instance, 80% of AD patients have insulin resistance or type 2 diabetes^111^ and diabetes increases the AD risk 2–5 fold compared to healthy individuals.^112^ Further diseases facilitating AD are cardiovascular diseases, traumatic brain injury, epilepsy, and depression.^113^ Again, these risk factors are associated with inflammation, oxidative stress, and carbonyl stress leading to extensive nPTMs.^114,115^

While modulation of APP cleavage by PTMs has been proposed, it has not been experimentally verified.^116,117^ Several amino acids next to secretase cleavage sites are prone to posttranslational modification (Fig. 3(B)). Recently, succinylation of APP residue K612 (=K16 in Aβ) was detected in 9 out of 10 brains from AD patients, but not in age-matched controls without dementia.^118^ This specific residue is the cleavage site of α-secretase and involvement of this PTM in shifting APP cleavage towards amyloidogenic cleavage comes to mind. Furthermore, the N-terminal amino acid in APP at the β-secretase cleavage site located right next to the Aβ 1–42 motif is M696. Oxidation of this site was investigated more than 25 years ago, but unfortunately the exact identity of β-secretase (= beta-site amyloid precursor protein cleaving enzyme 1) was unknown at this time, leading to tests with unsuitable proteases.^119^ The second β-secretase cleavage site of APP is next to Y10 of Aβ, which is readily nitrated and forms dityrosine crosslinks.^120,121^ In mice this site is preferred by β-secretase^122^ and leads to non-amyloidogenic Aβ 11–42.^105^

The Aβ 1–42 peptide itself is heavily modified at various sites (Fig. 3(C)). Aβ extracted from senile plaques is often oxidized at M35.^123^ Selective oxidation of M35 by H_2_O_2_ to methionine sulfoxide results in a threefold lower fibrillization rate compared to native Aβ.^124^ Neurotoxicity of native Aβ and M35 sulfoxide Aβ in primary cortical neuron cell culture is very similar,^125^ but replacement of M35 by norleucine significantly decreases toxicity in rat neuronal cells.^126^ Tyrosine oxidation leads to intermolecular dityrosine crosslinks between Y10 residues of Aβ and is commonly detected in AD brains.^120^ Dityrosine Aβ dimers are highly efficient in membrane permeabilization and exhibit significantly higher toxicity compared to wild-type Aβ.^127,128^ Hence, dityrosine formation is considered as a central mechanism in AD development.^129^ Nitroxidative stress causes nitration of Y10. A study involving *in vitro* nitration of Aβ found inhibition of fibrillization, increase of oligomerization, and consequently increased neuronal toxicity.^130^ The inhibition of Aβ aggregation by nitrotyrosine was further verified by dynamic light scattering experiments.^121^ Conversely, Kummer *et al.* measured increased aggregation of Aβ after Y10 nitration *in vitro* and reduced amyloid plaques as well as cognitive decline in an AD mice model after inhibition of nitric oxide synthetase 2.^131^ Senile plaques of AD patients contain Aβ adducts with lipoxidation product 4-HNE.^132^ However, 4-HNE only has minor if any effects on aggregation of Aβ1–40. Unfortunately, this study did not check the influence of 4-HNE on Aβ1–42 aggregation.^133^ Addition of 4-HNE to a neuroblastoma cell line is highly cytotoxic, but conditional expression of a C-terminal APP fragment has not further increased this effect.^134^ The Aβ peptide contains 3 aspartic acid residues at positions 1, 7, and 23, which are all prone to d-isomerization.^135^ The formation of d-aspartate is enhancing proteolytic resistance and aggregation of Aβ.^136^

Protein glycation in AD patients is up to three times higher than in control subjects and even further increased in Aβ plaques.^137^ MGO treatment decreases Aβ fibrilization kinetics,^138^ but increases Aβ aggregate size^133^*in vitro* and enhances toxicity of C-terminal APP fragment expressed in neuroblastoma cells.^134^ While the arginine modification MGO-derived hydroimidazolone 1 (MG-H1) is the most abundant AGE in cerebrospinal fluid of AD patients,^139^ MGO preferentially glycates K16 over R5 in Aβ.^140^ Synthetic Aβ incubated with MGO is more toxic for hippocampal neurons compared to unmodified Aβ and treatment of Tg2576 mice with dicarbonyl scavenger aminoguanidine ameliorated cognitive decline in this AD model.^141^ Solid phase peptide synthesis (SPPS) was used to site-specifically incorporate CEL modifications in Aβ at positions K16, K28, and double-mutation of K16 and K28.^142^ According to this study, K28CEL has no effect on fibrilization, while K16CEL slows fibril formation, and K16,28CEL alters the aggregate morphology. Nevertheless, K16CEL and K28CEL exhibit higher toxicity in differentiated SH-SY5Y cells compared to unmodified Aβ, whereas K16,28CEL completely loses its toxic properties.^142^ These findings clearly indicate that *in vitro* and in cell evaluation is required to assess nPTM effects, posing the challenge for all synthetic, modified peptide and protein samples to be transferred into *in vivo* systems in a sensible manner.

### Tau

**Fig. 4:**
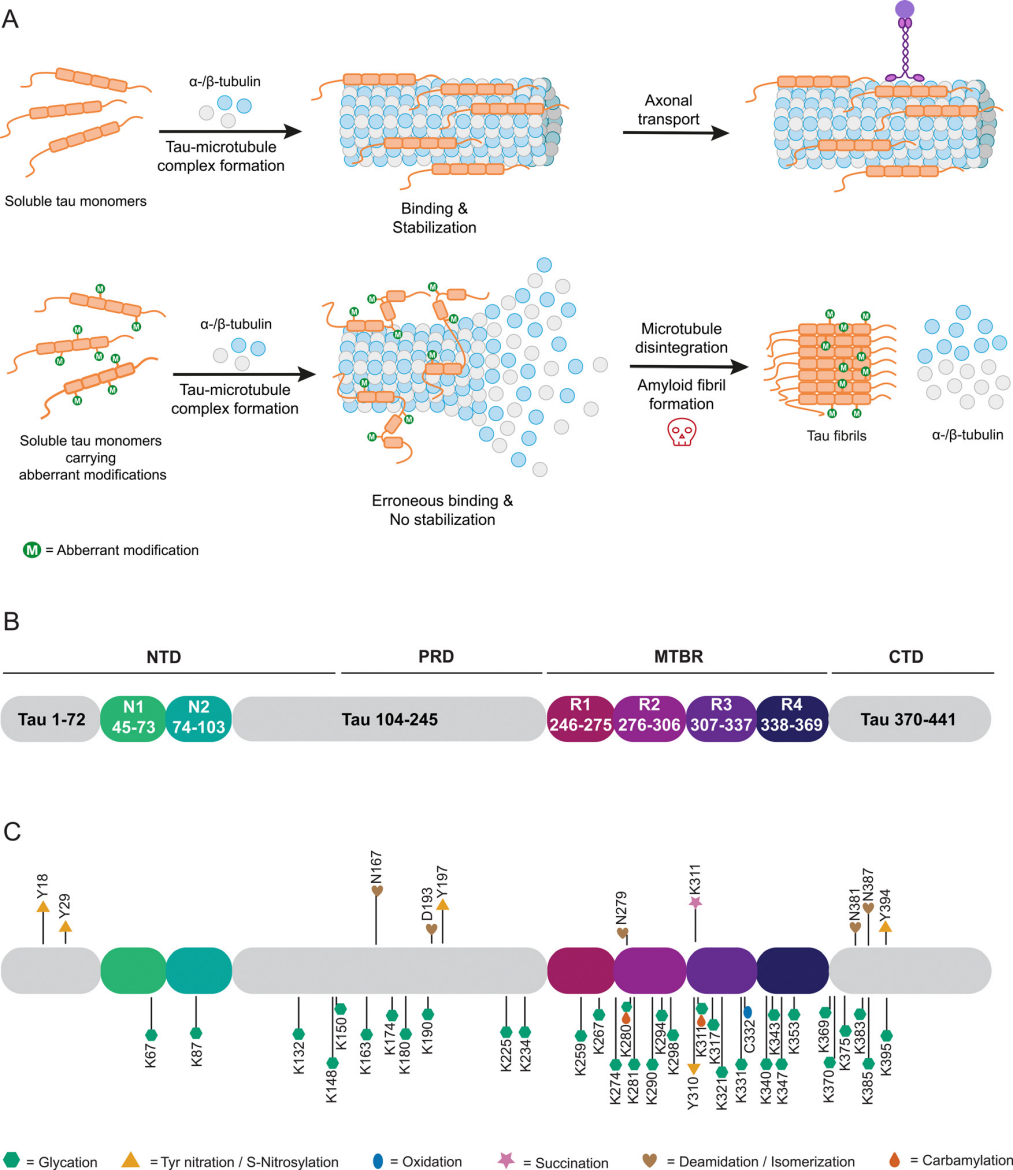
Overview of tau protein, physiological function and aggregation process, and known non-enzymatic posttranslational modification sites. Physiological and pathological function of tau in stabilization or disintegration of microtubule (A). Domain structure of tau 4R2N isoform (B). Non-enzymatic posttranslational modifications of tau 4R2N. *in vivo* nPTMs detected in samples above, *in vitro* generated nPTMs below (C).

The microtubule associated protein tau was first purified from porcine brain alongside tubulin.^143,144^ Further research has placed tau in the central and peripheral nervous system, being most abundant in neuronal axons.^145^ Tau is typically associated with the promotion of polymerization, assembly and stabilization of microtubules (Fig. 4(A)), thus contributing to the structural integrity of neurons and axonal transport.^146^ When present in other intracellular compartments or extracellular locations, tau exhibits additional functions such as protecting DNA from peroxidation-induced damage within the nucleus.^147^ Numerous other potential roles for tau remain under investigation.^148–152^

Structurally, tau belongs to the family of intrinsically disordered proteins and exhibits no preferred three-dimensional structure.^153^ Tau exists in six isoforms of varying length, differing at their N-terminal and microtubule-binding regions. Three varying N-termini derive from alternative splicing of exons 2 and 3, with either no N-terminal extension (0N), 29 (1N) or 58 (2N) additional amino acids present. Furthermore, tau isoforms may contain three or four repeated microtubule-binding domains (3R or 4R) by exclusion or inclusion of exon 10.^154,155^ Hence, making 4R2N the longest tau isoform, comprising of 441 amino acids. Intrinsically disordered proteins often act as scaffolds for signaling and regulatory functions, allowing for highly promiscuous interactions reflected in the number and diversity of potential tau functions. The 4R2N isoform of tau is characterized by a negatively charged N-terminus (amino acids 1–121), followed by a domain with a high excess of positive charge (122–250), and repeat regions (Fig. 4(B)) with a moderate excess of positive charge (251–390) as well as a negatively charged at the C-terminal domain (391–441). Based on sequence features and the N (N1, N2) and R (R1–R4) domains, 4R2N tau may also be subdivided into an N-terminal domain (NTD; 1–150), proline-rich domain (PRD; 151–243), microtubule binding region (MTBR; 244–368) and C-terminal domain (CTD; 369–441). Containing a large number of residues subject to PTMs, such as phosphorylation, acetylation or ubiquitylation, enables fine-tuning of tau's biological activity.^152,156^ Aberrant modifications, particularly hyperphosphorylation, are implicated in tau-related diseases, coined tauopathies. By disruption of native tau function, neuronal transport, axonal transport and stability are perturbed (Fig. 4(A)), making tauopathies neurodegenerative diseases, categorized by the disease-associated isoforms 3R, 4R, and 3R + 4R. Some of these include Alzheimer's disease (AD), amyotrophic lateral sclerosis, familial frontotemporal dementia, and Pick's disease.^152^ It is important to note that tau dysfunction may contribute to disease pathology, though it may not be the primary cause. A defining characteristic of tauopathies, first observed by Alois Alzheimer in the brains of AD patients in 1906, is the presence of insoluble inclusions composed of neurofibrillary tangles (NFTs) formed by tau protein.^157^ These NFTs consist of paired helical filaments (PHFs) of tau.^158,159^ Notably, tauopathies are distinguished by unique pathological filament structures, as the conformers vary between different diseases, although they remain consistent among patients with the same condition.^160,161^

The major focus of previous research was fibrillar tau in AD. Aggregated tau isolated from brain samples carries a specific pattern of phosphorylation different to that of healthy controls and is discussed in detail in several reviews.^162–165^ Furthermore, modifications such as acetylation and ubiquitylation have garnered increasing interest and have been studied by different approaches allowing controlled installation of PTMs in tau.^166–170^ However, tau aggregation can also be influenced by a variety of nPTMs, which will be discussed in the following.

Tau protein isolated from AD patients’ brains and in helical filaments is glycated (Fig. 4(C)).^171–173^ Non-specific *in vitro* glycation of tau by reducing sugars enhances aggregation of isoforms 4R2N and 3R2N and reduces tubulin binding.^174–176^ Combining non-specific phosphorylation and glycation of tau enhances aggregation.^177–179^ Such undefined glycation reactions can lead to tau variants glycated at up to 24 lysine residues (Fig. 4(C)).^175^ However, such ambiguous and most likely excessive glycation of a target protein does not reflect (patho-) physiological conditions and in turn does not allow understanding the impact of individual modifications. We have previously introduced the site-specific carboxymethylation of K294 into tau *via* protein semi-synthesis and found an inhibitory effect on tubulin polymerization, without directly impacting tau aggregation.^167^ In all likelihood, changes in tau properties induced by glycations are modulated by more specific glycation events controlled by concentration of electrophiles and accessibility of reactive side chains, arguably severely reducing the relevance of experiments carried out *via* unselective glycations. Here, indirect effects of nPTMs, for example on chaperone systems that should prevent protein aggregation, can also come into play as was recently demonstrated for argpyrimidine modifications of heat shock protein 27 (Hsp27). Activity of Hsp27 towards several client proteins was severely impacted by one or more argpyrimidine residues incorporated by protein semi-synthesis.^180^

Another prevalent nPTM found at elevated levels in brain isolates from AD patients is 3-nitrotyrosine.^181^*Via* nitrotyrosine specific antibodies, nitration at Y18, Y29 and Y197 was detected. While nitration of Y18 and Y197 is observed in AD brains and age-matched controls, nitration at Y29 appears to be specific for AD and Pick's disease.^182–184^ Y197 is also endogenously nitrated in mouse brain samples and PC12 cells as models for AD.^185^ Single-site nitration of Y18 inhibits tau aggregation *in vitro.*^182^ Effects of nitration were also evaluated *in vitro* through addition of RNS such as peroxynitrite. Based on these results, nitration may occur in hierarchical fashion with Y18 and Y29 being preferred over Y197 and Y394.^186^ However, the results of such artificial nitration conditions need to be carefully evaluated. Formation of larger tau aggregates is reduced for this variant with multiple nitrations, however, these modifications may induce oligomerization of tau.^187^

nPTMs can control peptide and protein aggregation as demonstrated for non-specific carbamylation of short peptide fragments of tau containing neighboring lysine residues. Such modified peptides strongly vary in their aggregation behavior with ^140^KKAKGA^145^ not aggregating, ^148^KTKIAT^153^ strongly aggregating, and ^224^KKVAVV^229^ showing medium aggregation levels. Additionally, ^254^KNVKSK^259^ aggregates at high concentrations or at low concentrations upon adding nonpolar residues and ^368^NKKIETHKLTF^378^ shows a concentration-dependent aggregation.^188^ Specific carbamylation of the N-terminally acetylated hexapeptides PHF6 (^306^VQIVYK^311^) and PHF6* (^275^VQIINK^280^) led to rapidly aggregating PHF6 and formation of fibrils that exhibit increased cytotoxicity, whereas PHF6* shows an extended lag-time and a slow, linear aggregation profile when acetylated and carbamylated. In the same study non-specific carbamylation of full-length tau was tested, resulting in a protein or protein mixture that aggregates quickly even in the absence of an external inducer that is commonly used in such *in vitro* aggregation assay to reduce measurement time and to increase reproducibility.^189^

Oxidative and reducing conditions modulate the cysteine thiol group. Here, several reports analyzed the impact of disulfide linkages or the oxidation state of cysteine on tau behavior. In summary, these findings implicate the disulfide bridge between C291 and C322 or intermolecular bonds in increased aggregation and polymerization of tau. Tau aggregates accumulate in fly retina and mouse primary cultured neurons under oxidative stress, which the authors link back to intermolecular disulfide bond formation with cysteine-deletions disrupting assembly.^190^ Aggregation kinetics were also shown to be increased under oxidizing conditions and with all cysteines intact. Alanine replacement of cysteine at position 291 or 322 reduces dimer assembly rate, an effect stronger for 2R and 3R than 4R isoform, assuming dimerization and polymerization of 3R to rely on C322 intermolecular disulfide formation, which in turn acts as polymerization seed. The disulfide linkage between 3R1N and 4R1N accelerates fibrillation kinetics.^191^ The oxidation state of C322 in particular appears to modulate tau-associated toxicity and dysfunction.^181,192^ Oxidation of 4R2N induces formation of structurally distinct fibrils, which could be resolubilized through the addition of reducing agents.^193^

An optional lysine PTM, which has garnered increasing interest, is succinylation. Despite hypotheses on the origin of this PTM conflicting, besides putative succinyl transferases, a non-enzymatic process is considered a likely cause, therefore, being detailed for tau in the following.^41,194^ Recently, first insights into the succinylation of lysine residue 311 were revealed, demonstrating that succinylated lysine occurs in nine out of ten AD patient's brain samples, however, not in healthy controls.^118^ Specific succinylation of the PHF6 peptide at K311 accelerates aggregation, whereas modification of K280 in PHF6* does not aggregate under the same conditions. Furthermore, treatment of tau K19 (comprising residues Q244-E372) with succinyl-CoA results in complete loss of function in microtubule binding assays. Lastly, specific succinylation of the tau 296–321 peptide at K311 decreases binding affinity for tubulin.^195^ Specific modification was achieved by SPPS.

**Fig. 5:**
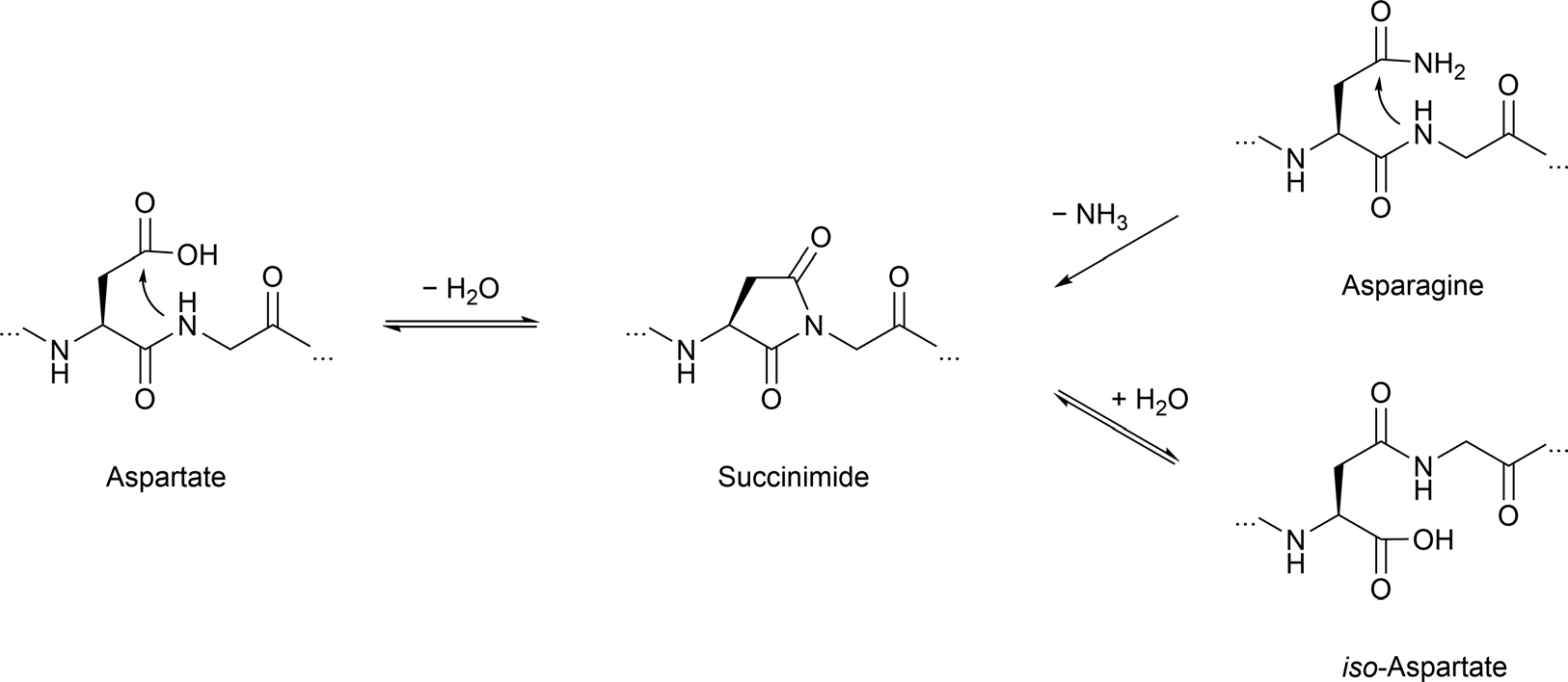
Asparagine deamidation and aspartate isomerization reaction proceeding *via* the succinimide intermediate.

Another commonly observed irreversible, non-enzymatic modification occurring during protein aging is deamidation of asparagine. A similar deamidation can be observed as a consequence of asparagine isomerization (Fig. 5), which may occur enzyme catalyzed or enzyme-free and which has also been observed for tau.^196,197^ This observation was triggered by broad bands in Western blots of tau PHFs.^198^ This notable feature was further investigated by analysis of the protein samples in these bands and revealed the presence of significant amounts of d-aspartate in tau.^196^ This finding has been confirmed by another study focusing on smeared bands found for PHF. Here deamidation and isomerization are found on residues N381 and N387 giving rise to isoaspartates.^199^ Another study detected isoaspartate at D193, N381 and N387 in PHF tau, supporting these findings.^200^ Employing antibodies specific for D387 and isoD387, the PHF smear was immunostained to a higher degree than PHFs, which led to the conclusion that the modifications mainly occur after fibril formation.^201^ This observation touches on a critical point when studying nPTMs as we still struggle with determining the sequence of events in forming nPTMs.

In contrast to this, deamidation can also lead to functional consequence as described for asparagine residue 279. In this case, Asn to Asp conversion negatively affects microtubule assembly.^202^ Intriguingly, extensive deamidation of 3R and 4R tau is exclusively observed in AD, yet neither progressive nuclear palsy or corticobasal degeneration. In order to study the molecular basis site-specific d-isomerization in tau R2 and R3, the main components of tau PHF core, peptide with d-Asp were generated by SPPS. These model peptides exhibit reduced time-dependent transition from random coil to β-sheet following heparin treatment compared to a wild-type R2 peptide.^203^ Another study employing synthetic R2 and R3 peptides respectively, demonstrates notable attenuation of the inhibitory effect of cyanidin on fibrillation for peptides containing d-isomerized aspartate. Additionally, peptides of this series show an altered fibril morphology.^204^ No synergistic effects for the double-isomerized peptide compared to the single isomerized variants are observed. Additionally, isomerization of serine in tau R3 affect β-sheet transition or fibril formation compared to the WT R3 peptide.^203^

### Prevention of protein aggregation by avoiding or repairing nPTMs

**Fig. 6:**
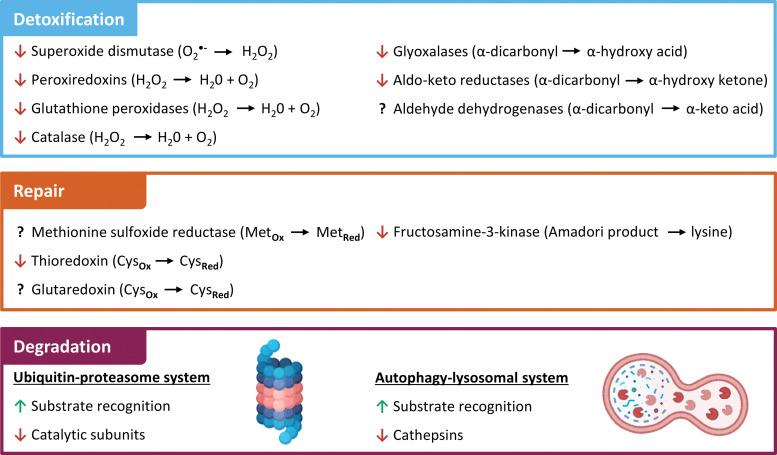
Effects of non-enzymatic posttranslational protein modification on cellular defense systems against protein aggregation. Effects of non-enzymatic posttranslational protein modifications on cellular detoxification, repair, and degradation systems are indicated by red arrows(inhibition), green arrows (activation), and question marks (unknown).

In the previous sections, we highlighted the importance of non-enzymatic posttranslational modifications (nPTMs) in the process of protein aggregation. Cells employ several enzymatic defense mechanisms to prevent accumulation of nPTMs (Fig. 6). The first line of defense involves the detoxification of potentially harmful electrophiles,^205^ which can arise from elevated levels of oxidative and carbonyl stress. The second line of defense focuses on the repair of mildly damaged proteins. Last but not least, severely damaged proteins are degraded *via* different pathways.^205^ Although these systems are crucial for preventing accumulation of proteins damaged by nPTMs, the enzymes involved in these processes are themselves susceptible to modifications. The following sections will summarize the known nPTMs in these enzyme systems and the resulting effects as a contributing factor in protein aggregation.

#### Detoxification

One of the main sources of reactive oxygen species (ROS) is superoxide, which is formed by leaking electrons in the mitochondrial electron transport chain. Superoxide is primarily converted by mitochondrial manganese superoxide dismutase (SOD2) to hydrogen peroxide.^206^ SOD2 nitration was reported as a consequence of inflammation in humans.^207^ Site-specific incorporation of 3-nitrotyrosine replacing the active center Y34 *via* genetic code expansion using an orthogonal aminoacyl-tRNA synthetase/tRNA pair results in a 97% loss of activity.^208^ Alternatively, superoxide is detoxified in the intermembrane space by copper/zinc superoxide dismutase (SOD1), which is strongly regulated by posttranslational modifications.^209^ Nitration of SOD1 by peroxynitrite at W32 decreases its activity by 30%^210^ and oxidation causes SOD1 aggregation.^211^ The detoxification of ROS by SOD is impaired by glycation.^212^ Incubation by MGO glycated arginine residues 69, 79, and 143^213^ and glucose modified lysine residues 3, 9, 30, 36, l22, and l28,^214^ both lead to reduced SOD1 activity. Glycation is also a possible cause for SOD1 aggregation in Amyotrophic Lateral Sclerosis as shown in yeast expressing hSOD1 and lacking glyoxalase 1 for MGO detoxification.^215^ The succinylation of K122 has no effect on SOD1 activity, but suppresses the ability of SOD1 to inhibit mitochondrial metabolism as proven by K122E mutation in cells.^216^ Multiple acylations ameliorated SOD1 seeding capability and thereby prevented further protein aggregate formation.^217^

The predominant enzymes responsible for elimination of hydrogen peroxide in cells are peroxiredoxins (Prx), glutathione peroxidases (GPx), and catalase. Their activities are regulated by a plethora of enzymatic modifications,^218^ but studies about the influence of nPTMs are rather scarce. Nevertheless, a shift from peroxidase to chaperone activity was reported after hyperoxidation of cysteine residues in Prx.^219^ Non-specific glycation by hyperglycemic conditions inhibits catalase^220^ as well as Gpx^221^ activity *in vitro*.

Short-chained dicarbonyls such as GO and MGO are mainly detoxified by the glutathione dependent glyoxalase system.^222^ The activity of this fundamentally important system is decreased in aged brains.^223^ Glyoxalase 1 is inactivated by nitration in cell culture^224^ and glutathionylation of C139 inhibits the enzyme.^225^ In tissues with low glyoxalase expression levels and in case of complex carbonyls, the detoxification is catalyzed by NADPH dependent aldo-keto reductases or aldehyde dehydrogenases.^226^ Elevated ROS levels are reported to cause cysteine oxidation and decrease enzymatic activity of aldo-keto reductases.^25,226^ Another example is the Parkinson disease protein 7 (DJ-1) that has various cellular functions such as transcription regulation, antioxidative modulation, and chaperone activity.^227^ DJ-1 scavenges glycated aSyn^228^ and was postulated as a potential deglycase.^229^ However, DJ-1 primarily counteracts glycation through its glyoxalase activity, reducing free MGO levels and shifting the equilibrium away from reversibly protein-bound MGO.^230^ DJ-1 is closely related to development of PD and point mutations^231^ or oxidative modification of C106 promote aSyn aggregation in PD patients.^232^

#### Repair

Mild oxidative damage at methionine and cysteine residues is reversible by enzymatic repair systems.^205^ One of the most important repair systems are methionine sulfoxide reductases (Msr), which reverse sulfoxide formation at methionine residues. At the same time the Msr system allows methionine residues to reversibly scavenge ROS and utilize their antioxidant potential to prevent more severe oxidative protein damage.^99,233^

Oxidized cysteine residues and unnatural disulfide bonds are repaired by the NADPH dependent thioredoxin–thioredoxin reductase system (Trx–TrxR system). Known target proteins are Msr and Prx.^234^ Trx itself is inactivated by oxidation of active site C33 and C35, which can be reactivated by the selenoprotein TrxR.^235^ However, oxidation of Trx at positions C62, C69, and C73 is not reversible by TrxR and resulted in a loss of activity.^236^ Alternatively, glutathione dependent glutaredoxin (Grx) reduces cysteine oxidation and removes glutathionylation.^237^ While the treatment of epithelial cells with MGO results in loss of Trx activity, the Grx is unaffected in contrast.^238^

Early intermediates in the glycation reaction cascade such as the Amadori product between lysine residues and glucose can potentially be repaired by fructosamine-3-kinase (FN3K). The enzymatic phosphorylation at the 3-hydroxy position of the Amadori product destabilizes the ketoamine leading to non-enzymatic breakdown into the native lysine and 3-deoxyglucosone.^239^ This protein deglycase is inactivated by oxidative dimerization at C32.^240^ Altogether, the presence of repair mechanisms for nPTMs emphasize the relevance and potential threat of nPTMs for protein homeostasis. In case repair of modified proteins fails, nPTM carrying proteins that have been rendered non- or malfunctional must be degraded.

#### Degradation

Such damaged proteins are either degraded by the ubiquitin-proteasomal system (UPS) or the autophagy-lysosomal system (ALS).^241^ The UPS has two primary degradation pathways. The first involves the ATP-dependent 26S proteasome, which targets and degrades polyubiquitinated substrates and plays a crucial role in the degradation of a wide array of regulatory proteins. The second pathway is the ATP-independent 20S proteasome, which is responsible for the degradation and clearance of damaged proteins.^242^ These oxidized and unfolded substrates lead to preferred recognition and degradation by the 20S proteasome.^243^ While the 26S proteasome is very susceptible for inactivation by oxidation, the 20S proteasome tolerates significant damage before its function is impaired.^244^ For instance, glutathionylation significantly decreases 26S proteasomal activity, while 20S activity is not affected.^245^ Overall proteasomal activity is reported to decrease after non-specific modification by 4-HNE.^246^ Incubation of epithelial cells with high glucose concentrations and induction of diabetes in mice decreases proteasomal activity without changes in expression levels.^247^

The UPS is unable to degrade cross-linked and small protein aggregates.^243^ Hence, the ALS is the last clearing pathway for these early species of protein aggregation.^248^ Similar to degradation by the UPS, moderate substrate unfolding by nPTMs increases accessibility for higher degradation levels and the ALS pathway is upregulated in stressed cells, but at some point key enzymes in ALS are also affected by oxidative damage.^249^ As an example autophagy-related protein 4 (ATG4), which is an important initiator of the autophagy cascade, is reversely inhibited by cysteine oxidation.^250^ Heat-shock cognate 70 (Hsc70), which is required for substrate detection in chaperone mediated autophagy, loses its chaperone activity by glycation.^251^ Finally, central proteases of the ALS such as cathepsins are reportedly inhibited by GO/MGO glycation in cell lysates.^252^

## Discussion

We have summarized here the effects of nPTMs on key proteins involved in neurodegeneration as described to date. A significant lack of molecular details becomes apparent when comparing the known facts on such nPTMs with “regular” enzyme-mediated PTMs. Therefore, many studies are merely descriptive and rely on identifying (and quantifying) nPTMs with antibodies against such modification and more recently on proteomics studies. The latter generate large datasets that need to be rigorously analyzed to identify physiologically relevant nPTMs but do not contribute to deciphering the molecular basis of how nPTMs effect protein function. A still unanswered question is the extent to which nPTMs and enzymatic modifications, such as lysine glycation *versus* enzymatic ubiquitylation, compete for the same modification sites and influence each other. The absence of precise model systems and analytical probes has significantly limited our ability to establish robust connections between oxidative stress, elevated levels of reactive metabolites, and protein aggregation. Hence, addressing these critical points is vitally important in future studies.

Many of the “mechanistic” studies on proteins carrying nPTMs cited here generate samples under highly artificial *in vitro* conditions based on incubation with glycating agent or oxidants at elevated temperature, with excessive reactant concentration in non-physiological solvents. In turn they mostly rely on *in vitro* measurement for example of protein aggregation *via* thioflavin T assay but do not aim at toxicological evaluations. Furthermore, most studies on nPTMs focus exclusively on *in vitro* systems, with limited efforts to validate these modifications or their effects *in vivo*. For example, the pathological relevance of nPTMs has rarely been verified using brain samples from patients with neurodegenerative diseases. Bridging this gap will require developing innovative tools to model nPTMs under physiologically relevant conditions and combining them with *in vivo* studies to evaluate their pathological impact.

Cell culture and *in vivo* approaches based on gene knock-outs of detoxification systems and/or genetic modifications inducing oxidative stress that both lead to an increase in nPTMs on proteins also suffer from a lack of molecular resolution and often complex reactions cascades. As an example, knock-down of glyoxalase 1 is a frequently used method to increase intracellular MGO concentrations. However, cells adapt their metabolism leading to mixed results, which makes it even more challenging to distinguish causes and effects of nPTMs.^253,254^

**Fig. 7:**
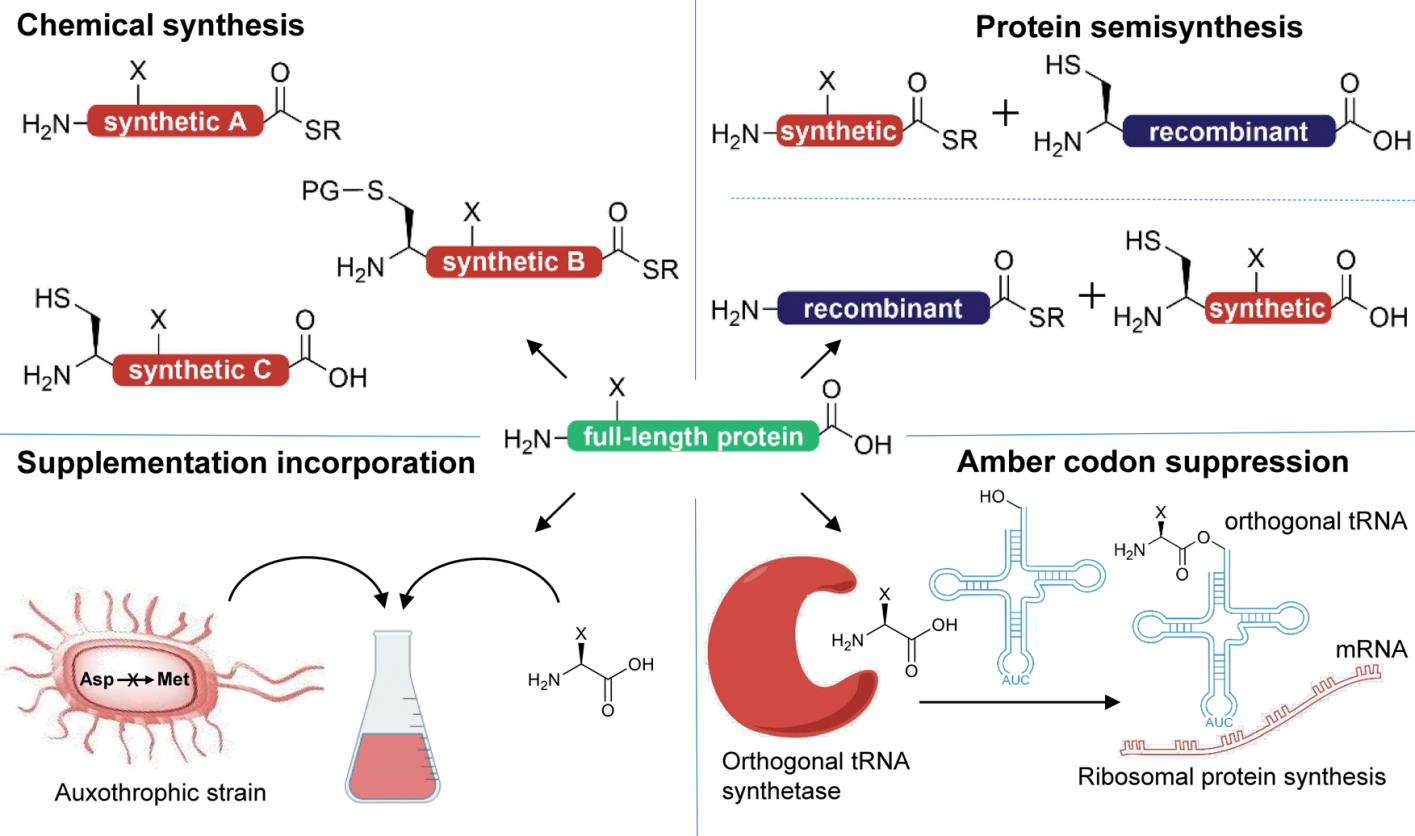
Methods for site-selective incorporation of non-enzymatic posttranslational protein modifications (indicated as X). Chemical synthesis *via* solid phase peptide synthesis in combination with chemoselective ligations reactions is the most flexible approach. Challenges related to protein size can be overcome by combing expressed protein segments with synthetic segments in protein semi-synthesis. To produce modified proteins in cells, exchange of one proteinogenic amino acid by supplementation of a structurally related non-proteinogenic amino acid is a viable method. This approach is exceeded by amber codon suppression in selectivity and flexibility (works in eukaryotic cells and expands the set of proteinogenic amino acids).

Obvious solutions to this dilemma can be found in approaches used to study enzyme-based PTMs such as depicted in Fig. 7. Chemical synthesis of peptides and proteins represents a direct way of addressing these questions by producing peptides and even proteins *via* chemoselective ligation approaches with nPTMs almost without any restrictions and with atomic precision.^255–257^ Currently this approach still suffers from the limited availability of many of the known nPTMs as useful building blocks for SPPS. Here, only few examples exist with robust synthetic access routes that either are long-known non-proteinogenic amino acids (*e.g.* citrulline) or have been more recently added to the available repertoire such as pentosinane and argpyrimidine.^258,259^

Another classic but often misleading approach has been copied from enzyme-based PTMs and is related to mutational exchange of proteinogenic amino acids, *e.g.* to suppress nPTMs such as nitrosylation on tyrosine by replacing it with phenyalanine.^260^ Such mutations have proven to be of limited use for PTMs (*e.g.* to mimic phosphoserine with glutamic acid) and we expect similar challenges when expanding this approach towards nPTMs. But we need to stress that mutational analysis by replacing reactive with unreactive amino acids (*e.g.* lysine to alanine mutations) remains a valuable tool.

Similarly, the exchange of sensitive residues such as methionine by norleucine (to exclude any oxidative damage) can be advantageous but requires the use of methionine-auxotrophic bacterial expression strains together with supplementation of norleucine (Fig. 7) and therefore additional levels of engineering.^261^ In a more flexible approach, amber stop-codon suppression (or more generally termed genetic code expansion) has been successfully used to site-selectively incorporate PTMs into a variety of proteins by relying on orthogonal pairs of tRNAs and tRNA synthetases that process specific non-proteinogenic amino acids and allow their incorporation *via* ribosomal protein synthesis.^262,263^ This approach could be quite easily expanded towards nPTMs if the modified amino acids are available together with suitable orthogonal pairs of tRNA and tRNA synthetases. It would be even more intriguing to combine such a code expansion approach with protein semi-synthesis as this allows modifying all parts of the protein of interest though with different flexibility.

In summary, more molecular details are needed to determine how nPTMs influence protein structure and function. Increasing insights into these mechanisms will demonstrate if intervention into processes leading to nPTMs can be exploited on a therapeutic level.

## Abbreviations

Aβ: Amyloid beta
AD: Alzheimer's disease
AGE(s): Advanced glycation endproduct(s)
ALE(s): Advanced lipoxidation endproduct(s)
ALS: Autophagy lysosomal system
APP: Amyloid precursor protein
ApoE: Apolipoprotein E
ATG4: Autophagy-related protein 4
CEL: *N* ^6^-Carboxyethyl lysine
CML: *N* ^6^-Carboxymethyl lysine
CTD: C-terminal domain
FN3K: Fructosamine-3-kinase
GO: Glyoxal
Gpx: Glutathione peroxidase
Grx: Glutaredoxin
4-HNE: 4-Hydroxy-2-nonenal
Hsc70: Heat-shock cognate 70 protein
Hsp: Heat-shock protein
MGO: Methylglyoxal
Msr: Methionine sulfoxide reductase
MTBR: Microtubule binding region
NADPH: Nicotinamide adenine dinucleotide phosphate
NFTs: Neurofibrillary tangles
NTD: N-Terminal domain
nPTM(s): Non-enzymatic posttranslational protein modification(s)
PD: Parkinson's disease
PHF: Paired helical filaments
PRD: Proline-rich domain
Prx: Peroxiredoxin
PTM(s): Posttranslational protein modification(s)
RACS: Reactive acyl-CoA species
RCS: Reactive carbonyl species
RNS: Reactive nitrogen species
ROS: Reactive oxygen species
sAPPα: soluble amyloid precursor protein α
SPPS: Solid phase peptide synthesis
SOD1: Copper/zinc superoxide dismutase
SOD2: Manganese superoxide dismutase
αSyn: α-Synuclein
Trx: Thioredoxin
TrxR: Thioredoxin reductase
UPS: Ubiquitin proteasomal system

## Data availability

No primary research results, software or code have been included and no new data were generated or analyzed as part of this review.

## Conflicts of interest

There are no conflicts to declare.
